# Multiple Primary Aneurysmal Bone Cysts: A Case Report and Literature Review

**DOI:** 10.7759/cureus.26509

**Published:** 2022-07-02

**Authors:** Ali H AlYami, Bandar N AlMaeen, Majed AlMuraee, AlWaleed A AlYami, AlBaraa AlGhamdi

**Affiliations:** 1 Department of Surgery, Ministry of the National Guard - Health Affairs, Jeddah, SAU; 2 Department of Surgery, King Abdullah International Medical Research Center, Jeddah, SAU; 3 Department of Surgery, King Saud Bin Abdulaziz University for Health Sciences, Jeddah, SAU; 4 Department of Surgery, College of Medicine, Jouf University, Al-Jouf, SAU; 5 Department of Orthopedic Surgery, King Faisal Specialist Hospital and Research Centre, Riyadh, SAU; 6 Department of Orthopedic Surgery, Ministry of the National Guard - Health Affairs, Jeddah, SAU

**Keywords:** clinical pathology, orthopedic oncology, giant cell tumor, metachronous, multiple aneurysmal bone cysts

## Abstract

Herein, we report the case of a 12-year-old boy diagnosed with multiple aneurysmal bone cysts (ABCs) who had previously undergone surgery on the proximal left tibia, proximal left femur, and distal tibia. During follow-up after the surgery, he developed another lesion on the proximal left humerus. Although rare, the pathological diagnosis was multiple ABCs.

## Introduction

Lichtenstein and Jaffe first used the term aneurysmal bone cysts (ABCs) indicating a distention of the affected bone with a gross appearance of a cavity containing fluid and blood [1]. ABCs are rare bone lesions accounting for 1−6% of bone tumors [2]. An incidence of 0.14 per 100,000 cases annually has been reported. The male-to-female ratio is approximately 1:1.04. The average age of presentation is 13 years, ranging from 1 to 59 years [3]. Clinically, ABCs tend to present more aggressively in younger patients [4].

Patients with ABCs usually present with pain, a palpable mass, pathological fracture, or a combination of these presentations. In a study of 238 patients with ABCs, the most common anatomical presentations were on the long bones, spine, or flat bones [5]. Radiographs indicate expansile lytic bone lesions with a “blown out” bony contour and a trabeculated appearance. Computed tomography (CT) scan shows fluid-fluid levels, and magnetic resonance imaging (MRI) indicates internal septations, fluid-fluid cysts, and low-intensity signals around the lesion [6,7].

Treatment mainly involves surgery. It includes excision and occasionally embolization of the feeding vessels. Curettage is the most commonly used modality of treatment. An en-bloc resection is an option when there is an active lesion or a recurrence. Selective embolization is used in surgically difficult anatomical locations or as an adjuvant [5]. Although the lesion is benign in nature, it tends to recur. Cases treated with curettage have a significantly higher rate of recurrence (p < 0.01) and confirmed incomplete excision is a risk factor for recurrence [3]. A clinicopathological study of 105 ABC cases reported a recurrence rate of 30.5% among all cases, with recurrence usually during the first two years after surgery [4,5]. Reports of multiple primary lesions of ABCs are even more infrequent in the literature. We only found four cases reported in humans [8-11] and two reported in animals [12,13].

## Case presentation

A 12-year-old boy with a diagnosis of multiple ABCs previously underwent surgery at another center on the distal left tibia and proximal left femur. He presented to our institute with a lesion on his left proximal tibia. After almost a year, he developed another lesion on his proximal left humerus. He had a history of three surgeries, with two being performed at another hospital.

**Figure 1:**
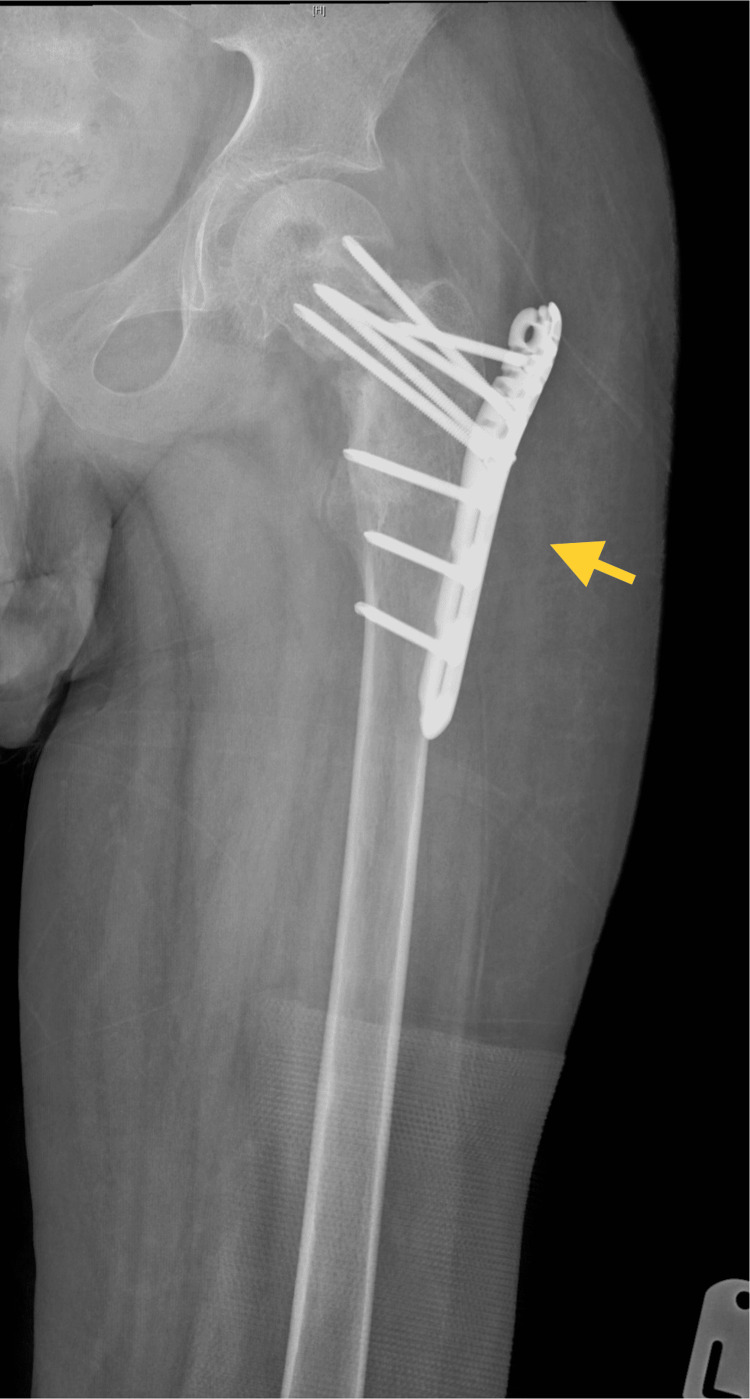
Postoperative anteroposterior radiograph showing the left femoral neck pathological fracture fixation.

The first lesion started at the age of eight on the left distal tibia. It was diagnosed as an ABC based on a pathological report at another health institution. The patient was treated with curettage and bone graft. He was followed up and no recurrence was noted. Approximately six months later, the patient had a pathological fracture of the left femur neck, which was managed by open reduction and internal fixation (Figure 1).

**Figure 2:**
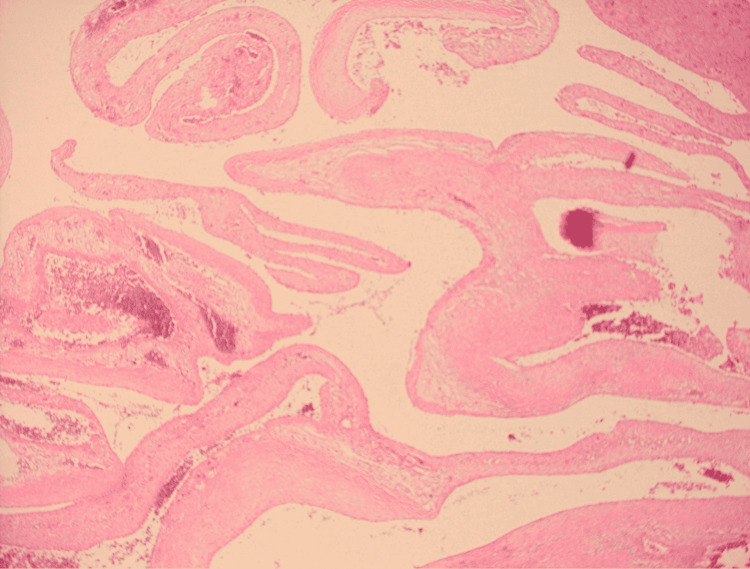
Microscopic picture of a pathology specimen taken from the left femur neck lesion.

Pathology specimens indicated fibrous tissues admixed with solid areas, new bone formation, giant cell reaction, and bloody spaces corroborating with the features of ABC (Figure 2). The patient improved with no significant sequel and no recurrence was noted.

**Figure 3:**
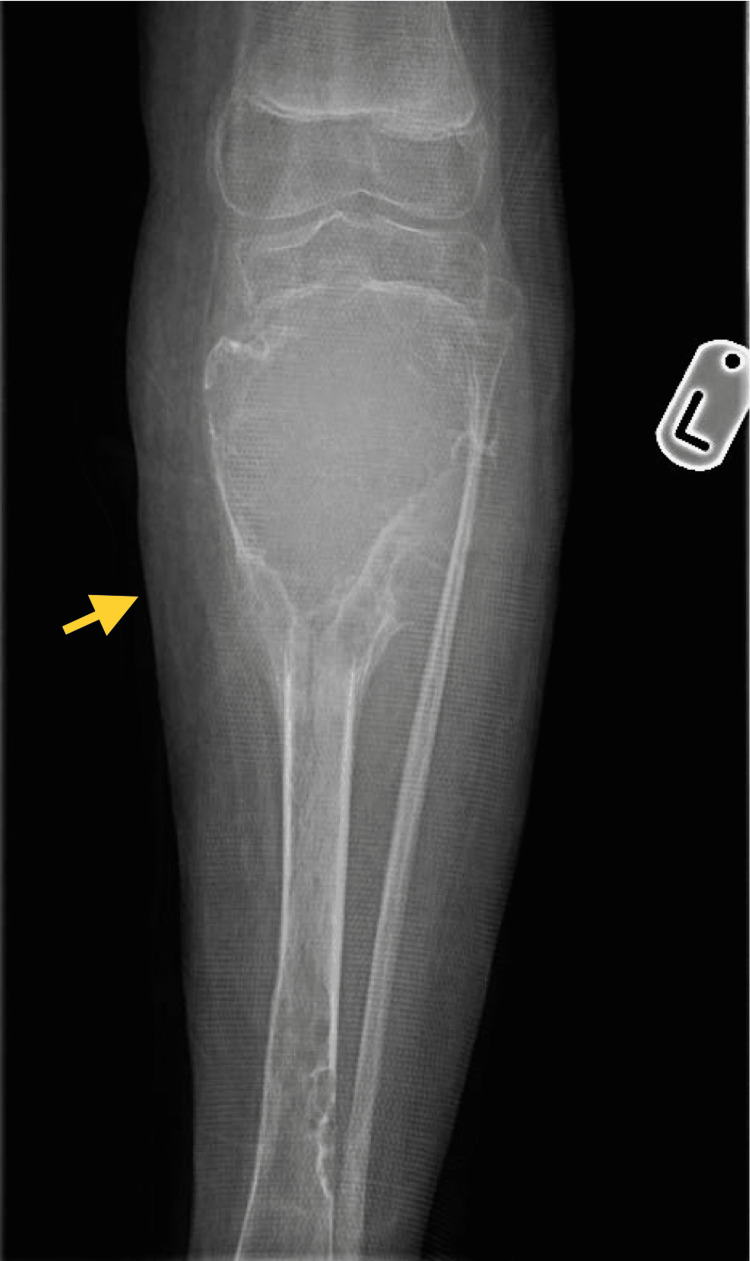
Anteroposterior radiograph showing a large aggressive expansile lytic lesion of the left proximal tibia. Another lesion was noted on the distal left tibia.

**Figure 4:**
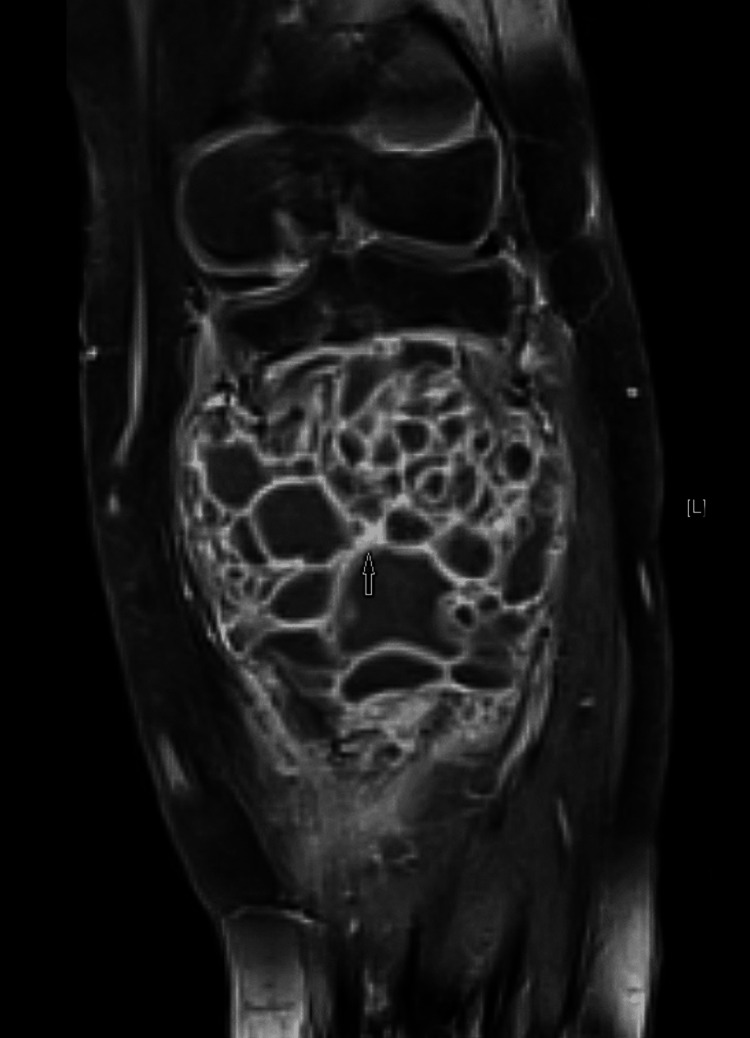
Post-contrast administration magnetic resonance images showing a large expansile lesion involving the proximal tibial metaphysis and diaphysis. This lesion demonstrated enhancement along the wall of the cystic changes and on the outline (arrow).

**Figure 5:**
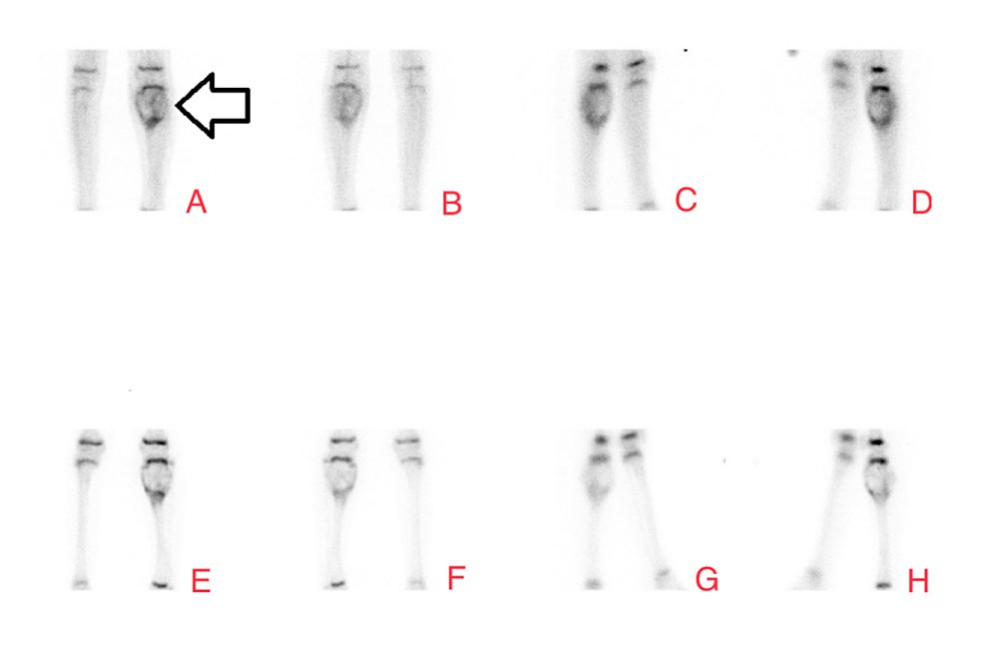
Tc-99m methylene diphosphonate bone scan flow images of both the knees. Tc-99m methylene diphosphonate bone scan flow images of both knees demonstrating an increased flow and hyperemia at the proximal aspect of the left tibia (arrow). Anterior (A), posterior (B), right lateral (C), and left lateral (D) views of the scan showing an increased flow and hyperemia at the proximal aspect of the left tibia after Tc-99m methylene diphosphonate injection. Anterior (E), posterior (F), right lateral (G), and left lateral (H) views demonstrating expansion of the left proximal tibia with increased uptake surrounding the known left proximal tibial lesion.

**Figure 6:**
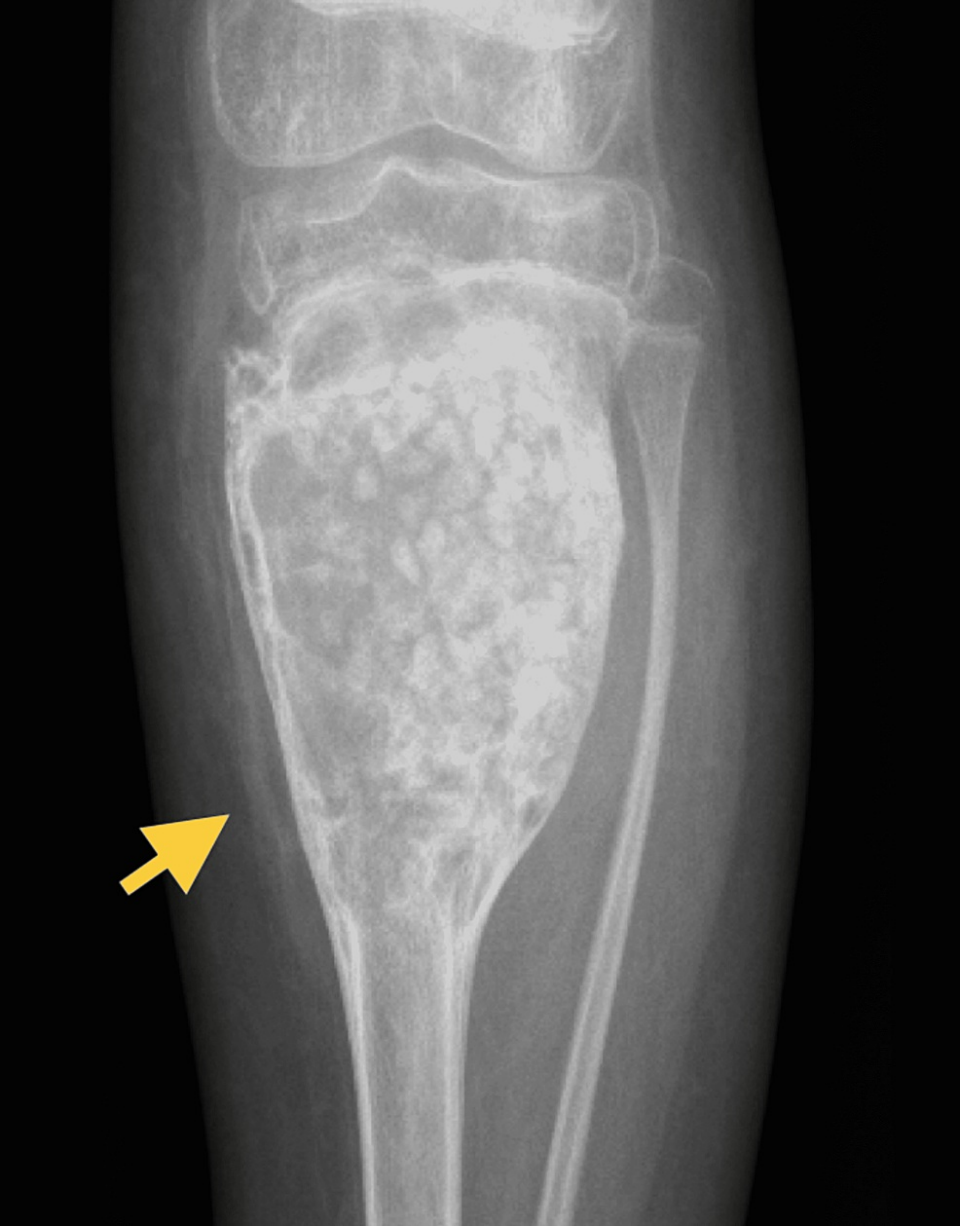
Postoperative anteroposterior radiograph showing an aneurysmal bone cyst on the left tibia treated with curettage and bone grafting.

Two years later, the patient presented with severe pain in the left leg, which was tender on examination. An X-ray showed a large cyst on the left upper tibia (Figure 3), suggestive of an aggressive bony lesion. An MRI showed that the lesion was highly consistent with an ABC (Figure 4). A bone scan showed an active and aggressive bone lesion involving the left proximal tibia in addition to a pathological uptake in the left proximal femur and left proximal humerus (Figure 5). The patient underwent surgery. Extensive curettage was performed accompanied by mechanical adjuvant burr therapy, and the cyst was filled with bone allograft (Figure 6). The final diagnosis resembled the features of an ABC. No malignancy was identified. Postoperatively, the patient recovered well. He was afebrile, vitally stable, and the wound was clean and dry. The patient was kept on prophylactic intravenous antibiotics and analgesics and was discharged home.

**Figure 7:**
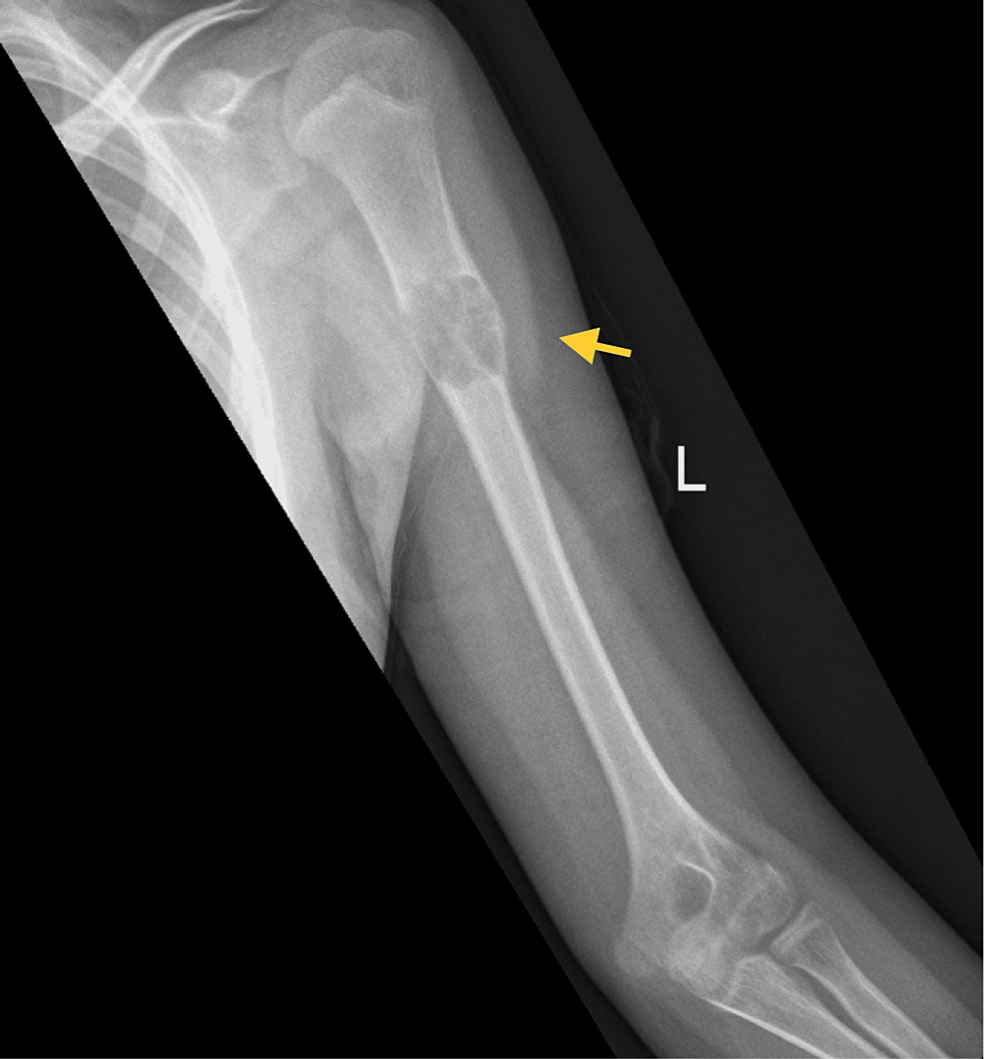
Anteroposterior radiograph showing a well-defined expansile lytic lesion at the proximal third of the left humeral shaft.

**Figure 8:**
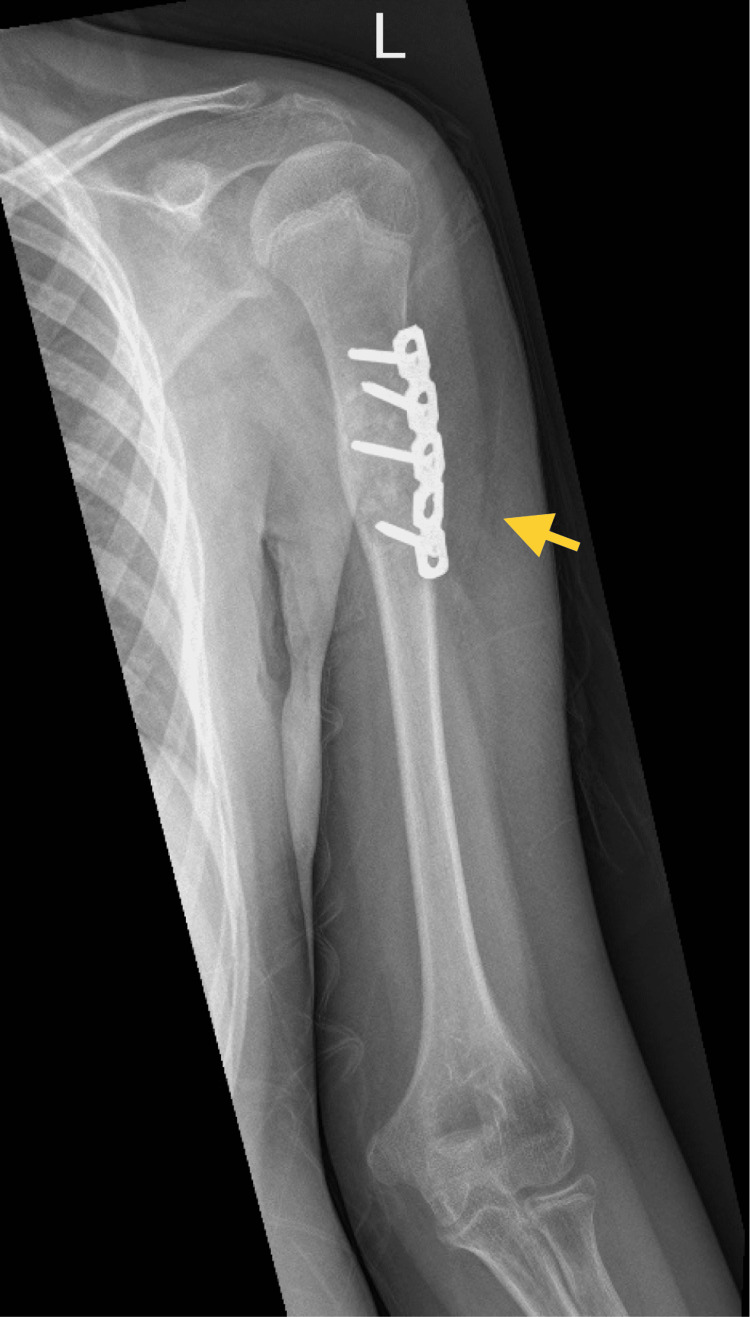
Postoperative anteroposterior radiograph of the aneurysmal bone cyst on the left humerus following curettage, bone grafting, and fixation.

**Figure 9:**
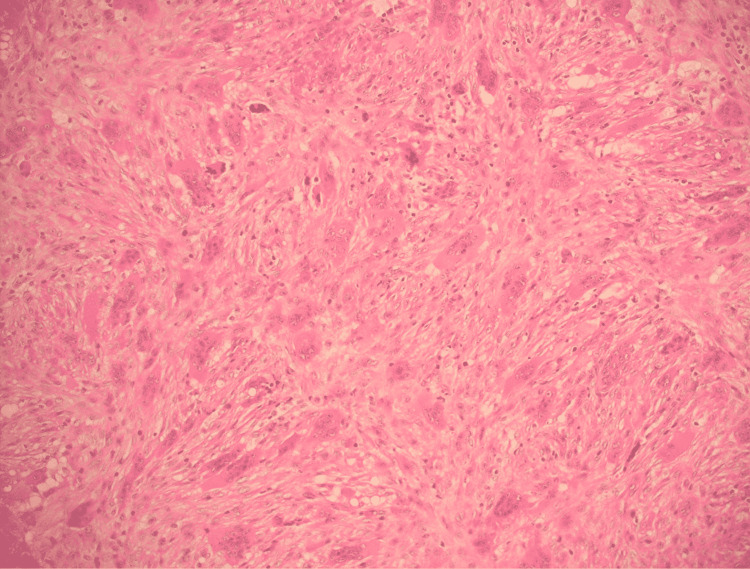
Microscopic image of the pathology specimen taken from the left proximal humerus lesion.

A year later, during the latest admission, the patient presented with pain and swelling of the left upper arm. An X-ray indicated a well-defined expansile lytic lesion on the proximal third of the left humeral shaft (Figure 7), which was managed with curettage, bone graft, and fixation of the left humerus (Figure 8). Based on the pathology report, a diagnosis of a giant cell-rich lesion consistent with a giant cell tumor was made (Figure 9). The postoperative period was uneventful. The patient was discharged home and followed up. He was doing well. There was no local recurrence. He was instructed to continue physiotherapy and was followed up every three months at the outpatient department. For two and a half years, he developed no signs of recurrence or any new lesion.

## Discussion

ABCs indicate a distention of the affected bone with a gross appearance of a cavity containing fluid and blood [1] Imaging including plain radiographs, CT scans, and MRI are diagnostic in the majority of the cases, but for definitive diagnosis histological examination of the lesion is required. Primary ABC histologically is a lesion with honeycombed dilated vascular beds with walls consisting of multinucleated cells, thin capillary vessels, granules of hemosiderin, and immature bone trabeculae [9]. Grossly, ABC manifested as a solid fleshy tumor. Differential diagnoses include giant cell tumor, telangiectatic osteosarcoma, fibrous dysplasia, simple bone cyst, osteoblastoma, and plasmacytoma [9].

Our patient was considered to have metachronous (multiple primaries) ABCs along with a recurrent ABC on a site other than the main site. This was supported by the bone scan findings as described. According to the best of our knowledge, this term has appeared only four times in the literature. A minimum of two ABCs are required for the diagnosis of “metachronous and multiple ABCs.” Our patient had four bone lesions; three were confirmed as ABCs and one giant cell tumor. It is known that giant cell tumors are the precursor lesions of ABCs, and they are sometimes mistakenly diagnosed as ABCs [1]. In ABC, giant cells are of smaller size and are unevenly distributed. In ABC solid type, the stroma is more fibrotic than that of giant cell tumor [14].

Our case is interesting because of the metachronous and multiple ABCs that are rarely found in the literature. All the primary lesions were located on the left side. No lesion was identified on the contralateral side. Similar scenarios were reported by Donigan and Sundaram [9].

The first of the four cases described in the literature was reported in 1997. Sundaram et al. described a case of ABC in a nine-year-old boy with a diametaphyseal osteolytic lesion on the left tibia. The lesion was curetted and packed with bone fragments. After 15 months, the patient returned with a new ABC on the left pubic region along with a recurrence of the ABC on the left tibia. Both the lesions had identical histological features. He was followed up for three years with no recurrence [8].

In 2003, Donigan et al. described a case of ABC on the left proximal humerus in a 17-year-old patient, which was treated by curettage. At the age of 32 years, he presented with a pathological compression fracture involving the left side of the eighth thoracic vertebrae (T8). An open biopsy confirmed the diagnosis of ABC, which was managed with an en-bloc resection at T7 and T8 along with reconstruction using a femoral allograft [9].

In 2004, Scheil-Bertram et al. described a case of a two-year-old boy with a history of congenital heart defects. He presented with a pathological fracture of the proximal humerus, which was diagnosed as ABC. Later, he developed another ABC affecting the right lateral clavicle, both distal radii, and right ulna. No genetic abnormalities were detected by cytogenetics [10].

In 2012, Amer et al. reported a case of a nine-year-old patient with a history of mild developmental speech delay and intermittent functional constipation since early childhood. The patient presented with a right scapular ABC, which was treated by percutaneous intralesional injection of doxycycline under fluoroscopic guidance. He had a second ABC affecting the left proximal tibial metaphysis 30 months later, which was treated with percutaneous intralesional chemoablation with a bone graft substitute [11].

## Conclusions

Multiple ABCs, although benign, have sequelae that could be debilitating to the patient. Here, we emphasize the importance of early diagnosis, appropriate treatment, and adequate follow-up of these lesions. For clinicians, it is important to consider multiple ABCs in the differential diagnoses in patients presenting repeatedly with swollen limbs and/or chronic bone pain.
